# Development of SCAR Primers for PCR Assay to Detect *Diplodia seriata*

**DOI:** 10.1155/2014/824106

**Published:** 2014-09-28

**Authors:** M. T. Martín, M. J. Cuesta, L. Martín

**Affiliations:** Instituto Tecnológico Agrario de Castilla y León-Zamadueñas, Carretera de Burgos Km 119, 47071 Valladolid, Spain

## Abstract

The aim of this study was to develop primer pairs for *Diplodia seriata* identification, one of the most common fungal species associated with grapevine decline in Castilla y León (Spain). Genetic variability of selected isolates of *D. seriata* was estimated. A molecular marker was generated from a random amplified polymorphic DNA (RAPD) fragment. PCR products of around 1200 bp were obtained with OPE20 primer. The PCR products were cloned and sequenced. The sequences were compared and a fragment of 1207 bp was used to design primer pairs. Two primer pairs were selected (DS3.8 S3-DS3.8 R6 and DS3.8 S3-DS3.8 R4) that amplified a single DNA product of 634 bp and 233 bp, respectively, with *D. seriata* isolates. No amplification was obtained for any of the 57 isolates of other species. The designed SCAR primer pairs allowed a rapid detection of *D. seriata*, and were able to detect 0.1 pg of the target DNA. Detection was specific and sensitive for *D. seriata*. The established protocols detected these fungi in naturally infected grapevines after DNA purification. *Diplodia seriata* was detectable without DNA purification and isolation in 62.5% to 75% of reactions. The detection of this pathogen in wood samples has great potential for use in pathogen-free certification schemes.

## 1. Introduction

Grapevine decline causes serious economic losses to the wine industry worldwide [1]. Among the numerous fungi associated with grapevine decline, species of Botryosphaeriaceae are dominant [2, 3]. They are well known as pathogens, saprophytes, and endophytes on a wide range of woody angiosperm and gymnosperm hosts [4, 5].

Identification of Botryosphaeriaceae spp. based on morphological characteristics remains a difficult task; therefore molecular sequence data and phylogenetic analysis are commonly used to confirm identifications [6–8]. Using these methods numerous species of Botryosphaeriaceae spp. have been identified and associated with grapevine decline worldwide, for instance,* Diplodia seriata*,* D. mutila*,* Botryosphaeria dothidea*,* Neofusicoccum parvum*,* N. mediterraneum*,* N. australe*,* N. ribis*,* N. luteum*,* Lasiodiplodia theobromae*,* L. crassipora*,* D. viticola* (*Spencermartinsia viticola*),* Dothiorella sarmentorum*, and* D. iberica* [1, 4, 5]. The development of restriction digest patterns following PCR amplification of the rDNA region has permitted the identification and detection of some Botryosphaeriaceae spp. [9]. Botryosphaeriaceae spp. were also identified based on sequencing of the ITS region although this sequence was not always sufficient to identify all species and sequences of other genes such as the elongation factor 1-alpha and beta tubulin which were required. These methods require the isolation of the fungi before DNA extraction and continue to be time-consuming and expensive.

Over the last two decades, newly established vineyards in Castilla y León (Spain) have shown an increasing percentage of plants with symptoms of wood decline. Research efforts allowed us to analyse grapevine samples from cankered and asymptomatic young and adult plants. Molecular methods were used to confirm the identification of* D. seriata*, which was one of the most abundant species found [2].

Molecular markers may be helpful in investigating numerous aspects of grapevine decline that still remain unclear, such as disease aetiology, epidemiology, taxonomy of the putative causal agents, and their genetic variability, and in improving diagnostic tools. In particular, random amplified polymorphic DNA (RAPD) markers were largely used for studying genetic variation in species such as* Erysiphe graminis* [10] and* Uncinula necator* [11]. RAPD markers were easy to perform and low in cost and no prior knowledge of the genome being investigated was required. RAPD markers also allowed the development of sequence characterised amplified region (SCAR) specific primer pairs, such as was published for* Monilinia* spp. [12] and* Agrobacterium vitis* [13]. In the case of grapevine decline Pollastro et al. [14] proposed SCAR primer pairs for the identification of* Fomitiporia mediterranea* and* Phaeomoniella chlamydospora* associated with esca and* Phomopsis viticola*, the causal agent of Phomopsis cane and leaf spot. Lardner et al. [15] published SCAR primers capable of amplifying DNA of* Eutypa lata*, the main causing agent of Eutypa dieback. The purpose of the present study was to develop SCAR specific primers, which could be used in conventional PCR to detect* D. seriata*.

## 2. Materials and Methods

### 2.1. Sample Analysis and Fungal Isolates

Fungi associated with grapevine decline were obtained from 381 vines. Fifty percent of the isolates were from Castilla y León, 47% from other Spanish regions, and 3% from other countries. Seventy-three percent were mature vine (>5 years). Sixty-four percent were grapevine branches. External symptoms were esca (22%), Eutypa dieback (34%), Petri disease/black food disease 2%, asymptomatic vines (19%), and others (23%). This study included 47* D. seriata*. Fifty-seven isolates of other species were used as controls. Tables 1(a) and 1(b) show the isolates' names and where they were obtained. Eleven cultures were obtained from the Centraalbureau voor Schimmelcultures (CBS Fungal Collection, Utrecht, The Netherlands),* Eutypella citricola* (810) was a kind gift from Dr. J. Luque (IRTA, Barcelona, Spain). All isolates were grown on potato dextrose agar (PDA) (Merck, Darmstadt, Germany) at 25°C in darkness. Fungal isolation, morphological and molecular identification, restriction patterns, DNA sequencing, and sequence analysis were done [2].

### 2.2. Extraction and Purification of DNA

For rapid detection, fungi grapevine genomic DNA was extracted using Redextract-N-Amp plant PCR kit (Sigma, St. Louis, MO, USA) following the manufacturer's instructions.

Genomic DNA was purified from mycelium using the DNeasy plant minikit (QIAGEN, Cologne, Germany). All DNA samples were diluted to a working concentration of 10 ng/*μ*L.

### 2.3. Development of SCAR Markers 

#### 2.3.1. RAPD Amplification

The PCR reaction mix was prepared following the commercial recommendation of Illustra GE Healthcare puReTaq Ready-To-Go PCR Beads (Amersham, Buckinghamshire, UK) supplemented with 1 mM of MgCl_2_ as described by [16], 0.7 *μ*M of primer, and 50 ng of DNA template in a final volume of 25 *μ*L. The PCR reaction was run according to [17]. Each run included as control a reaction without DNA. Each primer-template combination was tested at least twice. PCR amplifications were performed using a Gene Amp 7200 thermocycler (Applied Biosystems, Foster City, CA, USA) with the primers detailed below. After amplification, the total volume of the reaction of each PCR product was separated by electrophoresis in 1.5% agarose (low EEO, Conda Pronadisa, Madrid, Spain) gels in 1xTAE (Tris-acetate-EDTA). After electrophoresis (0.5 Vcm^−1^) the gels were stained with 0.5 *μ*g/mL ethidium bromide, visualized, and photographed using a UV transilluminator. A preselection of the 20 OPERON primers (Operon Technologies, La Jolla, CA, USA) of the OPA, OPD, and OPE series was made using the DNA of 23 isolates of* D. seriata*, five isolates of* D. mutila*, three isolates of* N. parvum*, one isolate of* B. dothidea*, and one isolate of* D. iberica*. Primers allowed patterns with distinguishable and reproducible bands to be preselected. Then fragment size and proximity of other bands were additional criteria for selecting the most specific marker for* D. seriata*. Each RAPD marker was treated and analysed [17].

#### 2.3.2. DNA Cloning

Eleven* D. seriata* isolates, Napa-c, CBS112555a, Y46-1-1b, Y62-1-1c, Y79-4-3a, Y90-10-1a, Y103-4-3a, Y112-24-1a, Y116-10-1c, V14-2a, and R21-1a (Table 1(a)), were used for cloning assays. The selected isolates represented the combination of the most variable geographic origin from our collection and different grapevine ages and included both isolates from both cankers and asymptomatic plants. OPE-20 RAPD markers specific for each isolate were separated by electrophoresis on 1.5% of agarose gel (TAE) and the 1200 bp fragment common to all* D. seriata* isolates was recovered, eluted, and purified using the commercial Kit GE Healthcare GFX PCR DNA and Gel Band Purification. DNA fragment was cloned by Sistemas Genómicos Agroalimentaria, Paterna, Spain. From each isolate five white/positive colonies of transformed* Escherichia coli*-DH5α were sequenced. The sequences were then analysed using the CLUSTALW (http://www.ebi.ac.uk/) program and SCAR primers were designed on the basis of the obtained sequences using http://www.ncbi.nlm.nih.gov/tools/primer-blast/.

### 2.4. PCR Amplification Using the SCAR Primers

#### 2.4.1. PCR Specificity

The specificity of each primer pair was tested in amplification tests with purified and/or extracted DNA of 47* D. seriata* isolates. Nontargeted DNAs of 57 isolates belonging to fungi associated with grapevine decline from our collection such as twelve Botryosphaeriaceae spp.:* D. mutila*,* B. dothidea*,* L. theobromae*,* D. sarmentorum*,* D. iberica*,* D. viticola*,* D. coryli*,* N. parvum*,* N. australe*,* N. mediterraneum*,* N. luteum*, and* N. ribis*, fourteen other fungi species:* P. chlamydospora*,* Phaeoacremonium aleophilum*,* Cylindrocarpon macrodidymum*,* C. liriodendri*,* C. olidum*,* C. pauciseptatum*,* P. viticola*,* E. lata*,* Eutypella citricola*,* Cryptovalsa ampelina*,* Cadophora luteoolivacea*,* Fomitiporia mediterranea*,* Fomitiporella coryophilli*, and* Stereum hirsutum*, isolates of other fungi belonging to the genera of* Fusarium*,* Alternaria*,* Acremonium*,* Didymella*,* Phomopsis*,* Epicoccum*,* Psathyrella*,* Ceratobasidium*, and* Pestalotia*, and* Vitis vinifera* cvs. Tempranillo and Viura were tested.

The PCR reaction mix was done following the commercial recommendation of Redextract-n-Amp plant PCR kit with the indicated modification: 4 *μ*L of the commercial mix, 0.4 *μ*M of each primer, and 1 *μ*L of template (purified or extracted DNA) in a final volume of 10 *μ*L. PCR reactions were then carried out according to the following basic scheme: the reaction mix was denatured at 95°C for 5 min, followed by 35 cycles of 30 sec at 94°C (denaturing), 45 sec at the annealing temperature of 57°C for DS3.8 S3-DS3.8 R6 or at 60°C for DS3.8 S3-DS3.8 R4, 45 sec at 72°C (extension), and a final extension phase of 7 min at 72°C. After amplification, the PCR products were separated by electrophoresis in 1.5% agarose gels in 1xTBE (Tris-borate-EDTA). The gels were stained and visualised as described above.

#### 2.4.2. PCR Sensitivity

The sensitivity of SCAR DS3.8 S3-DS3.8 R6 and DS3.8 S3-DS3.8 R4 primers was ascertained in PCR reactions with* D. seriata* CBS719.85 and Y207-1-1c. Assays were performed as three independent experiments. The following dilutions of DNA were used as template: 1, 0.1, 0.01, 10^−3^, 10^−4^, and 10^−5 ^ng/*μ*L. For these assays only purified DNA—DNeasy QIAGEN—was used.

#### 2.4.3. *D. seriata* Detection in Infected Wood Samples

Twelve* Vitis vinifera* (cv. Tempranillo grafted onto 110R-rootstocks) vines were inoculated with each of the* D. seriata* isolates Y103-4-2a and Y207-1-1c. 00E4 Twelve control plants were inoculated with a sterile agar plug (PDA). For the inoculation a wound was produced in the trunk of the vine, and an agar plug containing or not an actively growing culture of each isolate was placed on the wound and covered with parafilm. All grapevines were maintained in a greenhouse at 20–25°C. After four months, the* D. seriata* was reisolated and identified. These fungi could be reisolated 1 cm over the inoculation point and incipient wood symptoms could be observed. The effectiveness of the inoculation was evaluated using the conventional method of fungi isolation [2], consisting of cutting six wood chips (approx. diam. 1-2 mm; approx. length 0.5–1 cm) that were placed on malt extract agar plates-MEA (Merck, Darmstadt, Germany), and incubated at 25°C in darkness until fungi grew to an extent that could be isolated and placed on PDA plates. As above PDA plates were incubated and grown colonies were morphologically and molecularly identified using the primers pair described in this study (DS3.8 S3-DS3.8 R6 and DS3.8 S3-DS3.8 R4). The inoculated plants used in this study correspond to plants in which the inoculated fungi were isolated and identified from the six wood chips placed on MEA. Three wood chips were cut from four control plants and from four plants infected with each isolate. DNA purification was performed with one chip of each grapevine using the QIAGEN kit as described above. The second chip from each was placed in a tube containing 1 mL of malt extract (ME) medium, and the third chip was incubated in 1 mL sterile water. After 2 days at 25°C in darkness, conventional PCR was performed under the conditions described above with 2 *μ*L of purified DNA, 2 *μ*L of the incubation ME, or 2 *μ*L of the incubation water.

SCAR primers were validated under field conditions. Throughout field prospection eight samples of* Vitis vinifera* branches were excised from vines exhibiting black dead arm or Eutypa dieback symptoms. Seven samples were collected in Cigales and Toro (Spain) and one sample came from Portugal. Wood chips were excised from these naturally infected wood samples showing a dark area.* Diplodia seriata* was detected following the same procedure as described before for inoculated wood: fungi morphological identification, wood chip DNA purification, incubations, and PCR amplifications.

## 3. Results

Among the numerous species associated with grapevine decline, Botryosphaeriaceae spp. are the dominant with more than 540 isolates obtained from 381 samples. Molecular identification of 127 isolates of Botryosphaeriaceae using restriction patterns and ITS sequencing revealed that 65% of them were* D. seriata*.* Diplodia mutila* represented 6% and other species of Botryosphaeriaceae were identified in lesser percentages. All these methods are time-consuming, so, in order to improve the molecular identification, PCR specific primers were developed.

### 3.1. RAPD Analysis

The high resolution achieved with RAPD enabled the characterisation of intraspecific variation. In an initial screening, primers that provided reproducible patterns were selected; of the 20 primers in each of the OPA, OPD, and OPE kits, seven decamer primers were selected: OPA-2, OPD-2, -8, and -16, OPE-3, -19, and -20 to study single-spore cultures of the 23 selected* D. seriata* isolates, five* D. mutila*, three* N. parvum*, one* B. dothidea*, and one* D. iberica* and absence of DNA. Amplification with the selected primers provided 6–15 clear and reproducible bands. The product sizes obtained ranged from approximately 350 to 3000 bp. The combined results for the seven decamer RAPD primers produced 75 markers, generating ten genetically distinct groups of* D. seriata* isolates (data not shown) that indicated moderate genetic variation among the isolates studied in the present work.

**Figure 1:**
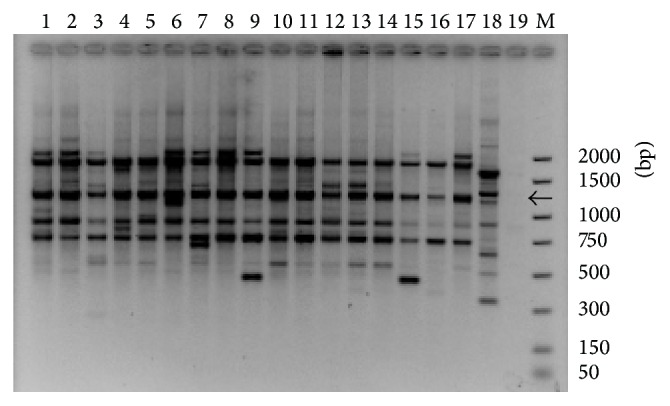
Random amplified polymorphic DNA of* Diplodia seriata* using primer OPE-20: amplified fragments were separated by 1.5% agarose gel electrophoresis (TAE). Lanes 1 to 17 corresponding, respectively, to* D. seriata* isolates: V14-2a, Y79-4-3a, Y103-3-3b, CBS719.85, Y84-1-1a, Y221-14-3a, Y213-8-2c, Napa-c, CBS112555, Y62-1-1c, Y90-10-1a, Y46-1-1a, Y112-24-1a, Y128-3-1b, Y168-21-1b, Y181-13-1b, and Y178-11-1a; lane 18:* D. mutila* Y50-7-2b; and lane 19: H_2_O. Lane M: DNA molecular weight marker; the fragment size is indicated on the right. RAPD marker OPE-1200 bp is indicated by an arrow.

Amplified products with OPE-20 generated four common fragments of about 1900, 1200, 960, and 795 bp and eight polymorphic fragments of about 2150, 1800, 1520, 1480, 1180, 870, 615, and 500 bp for* D. seriata*.* D. mutila* isolate gave a different pattern of bands and no band was amplified in absence of DNA template (Figure 1).* N. parvum*,* B. dothidea*, and* D. iberica* gave also different patterns (data not shown).

### 3.2. PCR Amplification Using the SCAR Primers

Species-specific markers common to all assayed isolates of* D. seriata* were searched. The OPE20-1200 bp fragment present in* D. seriata* isolates (indicated by an arrow in Figure 1) was selected. The 1200 bp fragment from eleven different* D. seriata* isolates (Table 1(a)) was cloned. Five positive colonies from each isolate were sequenced. Four fragments were obtained. A fragment (namely, DS3.8) of 1207 bp was selected. The sequence of the fragment DS3.8 was used to design five primers that were tested in five combinations. Each SCAR-primer pair was tested in an amplification experiment to establish appropriate operative conditions using DNA of* D. seriata*,* D. mutila*,* B. dothidea*,* N. parvum*,* L. theobromae*, and* D. sarmentorum* species present in Castilla y León and* N. ribis*. Afterwards three primers (DS3.8 S3 sense and DS3.8 R6 and DS3.8 R4 reverse) were selected and combined for conventional PCR: DS3.8 S3-DS3.8 R6 and DS3.8 S3-DS3.8 R4 producing 634 bp and 233 bp amplicons, respectively. Primer sequences are shown in Table 2. The experiments made it possible to select two primer pairs in the fragment DS3.8 which yielded the most reproducible results and a single band with strong fluorescence.

### 3.3. PCR Specificity

**Figure 2:**
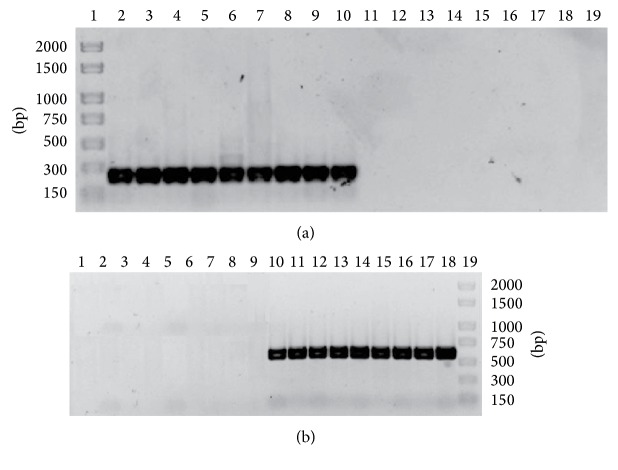
SCAR primers specificity. The amplified fragments were separated by 1.5% agarose gel electrophoresis (TBE). (a) The PCR amplifications were carried out using the primer pair DS3.8 S3-DS3.8 R4 yielding 233 bp amplicon. Lane 1: DNA molecular weight marker; the fragment size is indicated on the right. Lanes 2 to 10 corresponding, respectively, to* D. seriata* isolates: CBS719.85c, R21-1a, Y46-8-1b, Y63-4-1b, Y87-3-1c, Y111-27-1, Y116-27-96, Y121-15-4, and Y125-12-1b. Lanes 11 to 19 corresponding, respectively, to controls DNA of others species:* D. mutila* Y128-10-1,* N. parvum* Y159-24-1,* B. dothidea* CBS110302,* L. theobromae* CBS110.11,* D. sarmentorum* CBS120.41,* D. iberica* Y81-1-2,* N. luteum* CBS110299,* P. chlamydospora* CBS101359, and* P. aleophilum* CBS631.94b. (b) DS3.8 S3-DS3.8 R6 yielding 634 bp fragment. Lanes 1 to 9 corresponding, respectively, to controls DNA of others species:* D. mutila* Y128-10-1,* N. parvum* Y159-24-1,* B. dothidea* CBS110302,* L. theobromae* CBS110.11,* D. sarmentorum* CBS120.41,* D. iberica* Y81-1-2,* N. luteum* CBS110299,* P. chlamydospora* CBS101359, and* P. aleophilum* CBS631.94b. Lanes 10 to 18 corresponding, respectively, to* D. seriata* isolates: CBS719.85c, R21-1a, Y46-8-1b, Y63-4-1b, Y87-3-1c, Y111-27-1,Y116-27-96, Y121-15-4, and Y125-12-1b. Lane 19: DNA molecular weight marker; the fragment size is indicated on the left.

Both primer pairs produced a unique band of the expected size (634 bp and 233 bp) for 47* D. seriata* isolates using Redextract-N-Amp plant PCR kit (Table 1(a)). No specific fragments were obtained with any of the 57 isolates of 28 species other than* D. seriata* obtained and identified from symptomatic and asymptomatic grapevines. No specific fragments were obtained when* Vitis vinifera* was used as the DNA template.  Figure 2(a) showed the result of PCR amplification using DS3.8 S3-DS3.8 R4 primers with DNA of nine* D. seriata* isolates and nine DNA of other species (*D. mutila*,* N. parvum*,* B. dothidea*,* L. theobromae*,* D. sarmentorum*,* D. iberica*,* N. luteum*,* P. chlamydospora*, and* P. aleophilum*).  Figure 2(b) showed the result of PCR amplification using DS3.8 S3-DS3.8 R6 primers with DNA of the same isolates as before.

The specificity of the reaction was implemented using the DNA from other fungal species together (*D. mutila*,* N. parvum*,* D. sarmentorum*,* P. aleophilum*,* P. chlamydospora*,* C. pauciseptatum*, and* Alternaria* sp.) and the DNA of* D. seriata*: CBS719.85. The PCR conditions established for each primer pair produced only the expected amplicon (data not shown).

### 3.4. PCR Sensitivity

**Figure 3:**
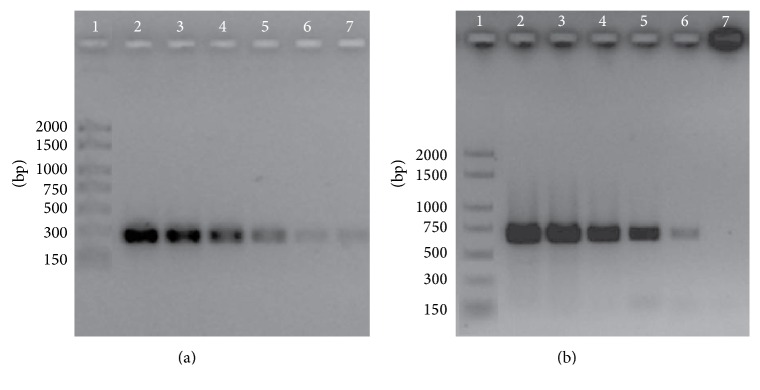
SCAR primers sensitivity. The amplified fragments were separated by 1.5% agarose gel electrophoresis (TBE). (a) The PCR amplifications were carried out using the primer pair DS3.8 S3-DS3.8 R4 yielding 233 bp fragment. (b) DS3.8 S3-DS3.8 R6 yielding 634 bp amplicon. Lane 1: DNA molecular weight marker; the fragment size is indicated on the right. Lanes 2 to 7 corresponding to* D. seriata* CBS719.85 as a template tested at decreasing concentrations: 1, 0.1, 0.01, 10^−3^, 10^−4^, and 10^−5^ ng/*μ*L, respectively.

The PCR efficiency for each primer pair was assayed with* D. seriata* purified genomic DNA. The 10-fold serial dilutions from genomic DNA were prepared from concentrated samples to obtain a broad range of dilutions (1, 0.1, 0.01, 10^−3^, 10^−4^, and 10^−5 ^ng/*μ*L). Each dilution was analyzed in triplicate with two different isolates. The serial dilutions of CBS719.85 genomic DNA were represented in Figure 3(a) using DS3.8 S3-DS3.8 R4 primers and Figure 3(b) with DS3.8 S3-DS3.8 R6 primers. A clear band remained visible in lane 5 for primers combination DS3.8 S3-R4 (233 bp) and in line 6 for primer combination DS3.8 S3-R6 (634 bp), establishing the detection limits in 1 pg and 0.1 pg of DNA, respectively.

### 3.5. Detection of* D. seriata* in Wood Samples

DNA purified from wood chips obtained from naturally infected grapevines or from grapevines inoculated with two different* D. seriata* isolates showed the expected 634 bp and 233 bp fragments using DS3.8 S3-DS3.8 R6 and DS3.8 S3-DS3.8 R4, respectively, for all samples. No amplification was observed with wood inoculated with a PDA plug containing no mycelium (Table 3). These results confirmed the above identification done using conventional methods consisting of the culturing of six wood chips in culture medium for each inoculated plant and morphological identification of the isolated fungi. Positive amplification of* D. seriata* in 62.5% to 75% of reactions was obtained with wood chips incubated for 2 days in culture medium with no DNA purification (Table 3). Field samples showing BDA and Eutypa dieback symptoms as well as grapevines inoculated with the Y103-4-2a or with Y207-1-1c isolate returned 634 bp and 233 bp fragments using DS3.8 S3-DS3.8 R6 and DS3.8 S3-DS3.8 R4, respectively. A positive reaction was found with three samples for each isolate and BDA symptoms and with two samples of plant showing Eutypa dieback. When incubation was performed in water for 2 days, the amplification result was lower (positive detection in 25% to 37.5% of reactions).

## 4. Discussion

Morphological identification of Botryosphaeriaceae spp. requires the work of specialists and time until the isolates produce spores that not always allow for the discrimination between genera and/or species. Based on the mycelium aspect, colour and growth on PDA medium 540 isolates from our collection were assigned as different species of the family Botryosphaeriaceae. Spores morphology helps in Botryosphaeriaceae spp. identification. However, nowadays molecular methods facilitate fungi identification. Sequencing and other molecular biology and PCR methods improve fungi identification, as well as studies of epidemiology and phylogeny. The development of restriction digest patterns following PCR amplification of the rDNA region has permitted the identification of some Botryosphaeriaceae spp. [9]. ITS sequence of* D. seriata* allowed the differentiation among different Botryosphaeriaceae spp. [9, 18–20]. However, it has not been possible to apply ITS alone to identify a Botryosphaeriaceae sp. Elongation factor 1-alpha and beta tubulin genes sequences allowed molecular differentiation among Botryosphaeriaceae spp. [21]. Restriction enzymes analyses and sequencing require manipulating the amplified products and increase the risk of contamination. Moreover they are time-consuming and more expensive than a conventional PCR with specific primers that reduce the identification steps in a single reaction.

Using restriction enzymes analyses and ITS sequencing methods the identification of 127 isolates of our collection was confirmed; 65% of them belong to* D. seriata* species. Preliminary RAPD enabled the characterisation of intraspecific variation of the most abundant Botryosphaeriaceae spp. found in Castilla y León grapevines. Taking into account all this information for the confirmation of the identification of the remaining isolates of our collection, SCAR primers were designed following the indication found in [10–14]. An OPE20 RAPD fragment of around 1200 bp was present in all* D. seriata* isolates and absent in the tested samples of* D. mutila*,* N. parvum*,* B. dothidea*, or* D. iberica*, so it was cloned. Among the cloned fragments a fragment of 1207 bp was selected and three primers allowed two combinations for conventional PCR. SCAR primers are available for easier identification of* D. seriata*.

The primers specificity was ascertained with the positive amplification of 47 isolates of* D. seriata* and the absence of specific fragments amplification with 57 isolates belonging to 28 different species. The sensitivity of the two PCR established the limit of* D. seriata* detection between 1 and 0.1 pg/*μ*L DNA. The sensitivity of SCAR primers published by Pollastro et al. [14] produced specific band with 0.1–1 ng of the target DNA. The PCR system described here improves and facilitates* D. seriata* identification. The easy and rapid detection of* D. seriata* will be an important advantage to guarantee the pathogen-free status of the propagated material in grapevine nurseries. The diagnostic methods described here improve previous techniques. To facilitate the diagnosis and taking into account our previous publication (Martín et al. [22]), wood chip samples were incubated in liquids. These liquids were then used as the DNA template in the PCR.* Diplodia seriata* PCR provided positive signals in 62.5% to 75% and 25% to 37.5% of the samples incubated in culture medium and water, respectively. To our knowledge, this is the first report of the use of conventional PCR for* D. seriata* detection in incubation liquid without DNA extraction and without a fungal isolation procedure. All isolates were 100% detected in wood chips after DNA purification by PCR and conventional isolation methods. More investigation is required to confirm these results. Detection of* D. seriata* without the need for fungal isolation reduces the analysis times to two days and reduces associated costs.

## 5. Conclusions

Two primer pairs named DS3.8 S3-DS3.8 R6 and DS3.8 S3-DS3.8 R4 were designed to perform a specific amplification of the pathogen* D. seriata*, one of the most common fungal species associated with grapevine decline. A single product of 634 bp and 233 bp, respectively, was obtained for* D. seriata* isolates but never from DNA of other 28 fungal species. A high sensitivity of the SCAR primers designed was found. These two conventional PCR were demonstrated to be useful in the specific detection of* D. seriata* on naturally and artificially infected grapevine wood without fungal isolation. Therefore a new simple, cheap, rapid, and specific diagnostic tool has been described for* D. seriata*. Moreover, the results of this study can be applied to other woody hosts for which this fungus has been reported as a pathogen.

## Figures and Tables

**(a):** 

Species	Strain	Location
*Diplodia seriata *	**CBS719.85c**	New Zealand
*D. seriata *	^*^ **N** **a** **p** **a**-**c**	California, USA
*D. seriata *	^*^ **C** **B** **S**112555**a**	Portugal
*D. seriata *	^*^ **Y**46-1-1**b**	Cigales, Spain
*D. seriata *	**Y46-8-1b**	Cigales, Spain
*D. seriata *	^*^ **Y**62-1-1**c**	Arribes, Spain
*D. seriata *	**Y63-4-1b**	Arribes, Spain
*D. seriata *	^*^ **Y**79-4-3**a**	Ribera del Duero, Spain
*D. seriata *	**Y84-1-1a**	Toro, Spain
*D. seriata *	**Y87-3-1c**	Arribes, Spain
*D. seriata *	Y87-8-1a	Arribes, Spain
*D. seriata *	^*^ **Y**90-10-1**a**	Navarra, Spain
*D. seriata *	**Y103-3-3b**	Extremadura, Spain
*D. seriata *	^*^ **Y**103-4-3**a**	Extremadura, Spain
*D. seriata *	Y111-27-1	Arribes, Spain
*D. seriata *	^*^ **Y**112-24-1**a**	Arribes, Spain
*D. seriata *	^*^ **Y**116-10-1**a**	Arribes, Spain
*D. seriata *	Y116-27-96	Arribes, Spain
*D. seriata *	Y121-15-4	Arribes, Spain
*D. seriata *	Y122-44-91	Arribes, Spain
*D. seriata *	Y125-12-1b	Arribes, Spain
*D. seriata *	Y126-73-91	Arribes, Spain
*D. seriata *	Y126-83-91	Arribes, Spain
*D. seriata *	**Y128-3-1b**	Galicia, Spain
*D. seriata *	Y161-1-1	Alicante, Spain
*D. seriata *	Y163-11-1	Alicante, Spain
*D. seriata *	Y163-13-1a	Alicante, Spain
*D. seriata *	**Y168-21-1b**	Alicante, Spain
*D. seriata *	Y169-11-1	Alicante, Spain
*D. seriata *	Y169-18-1	Alicante, Spain
*D. seriata *	Y169-18-2	Alicante, Spain
*D. seriata *	Y170-8-1	Alicante, Spain
*D. seriata *	Y171-3-1	Alicante, Spain
*D. seriata *	Y172-12-1	Alicante, Spain
*D. seriata *	Y172-19-3	Alicante, Spain
*D. seriata *	Y173-14-1	Alicante, Spain
*D. seriata *	Y173-17-1	Alicante, Spain
*D. seriata *	Y178-1-1	La Rioja, Spain
*D. seriata *	**Y178-11-1a**	La Rioja, Spain
*D. seriata *	Y180-20-1	La Rioja, Spain
*D. seriata *	Y181-9-1	Tierra de León, Spain
*D. seriata *	**Y181-13-1b**	Tierra de León, Spain
*D. seriata *	Y181-21-1	Tierra de León, Spain
*D. seriata *	**Y213-8-2c**	Córdoba, Spain
*D. seriata *	**Y221-14-3a**	Rueda, Spain
*D. seriata *	^*^ **V**14-2**a**	Nursery, Spain
*D. seriata *	^*^ **R**21-1**a**	Nursery, Spain

**(b):** 

Species	Strain	Location
*Diplodia mutila *	CBS431.82	The Netherlands
*D. mutila *	**Y50-5-1c**	Ribera del Duero, Spain
*D. mutila *	**Y50-7-2b**	Ribera del Duero, Spain
*D. mutila *	**Y60-7-2a**	Tierra de León, Spain
*D. mutila *	**Y63-1-1b**	Arribes, Spain
*D. mutila *	Y113-7-1	Arribes, Spain
*D. mutila *	**Y117-10-1b**	Arribes, Spain
*D. mutila *	Y122-10-1	Arribes, Spain
*D. mutila *	Y167-9-1	Alicante, Spain
*Neofusicoccum parvum *	CBS110301a	Portugal
*N. parvum *	**INIA_352c**	Madrid, Spain
*N. parvum *	**Y57-8-1b**	Nursery, Spain
*N. parvum *	**Y91-3-1a**	Nursery, Spain
*N. parvum *	Y108-9-1	Extremadura, Spain
*N. parvum *	Y159-24-1	Castilla La Mancha, Spain
*N. parvum *	Y187-8-1	Ribera del Duero, Spain
*Botryosphaeria dothidea *	**Sonoma_c**	California, USA
*B. dothidea *	CBS110302	Portugal
*B. dothidea *	Y264-19-1	Nursery, Spain
*Dothiorella iberica *	**Y51-4-3a**	Tierra de León, Spain
*D. iberica *	Y81-1-2	Ribera del Duero, Spain
*D. iberica *	Y190-3-3	Ribera del Duero, Spain
*Dothiorella sarmentorum *	CBS120.41	Norway
*D. sarmentorum *	Y51-4-3b	Arribes, Spain
*D. sarmentorum *	Y262-12-1	Nursery, Spain
*Lasiodiplodiatheobromae *	CBS110.11	nd
*L. theobromae *	Y512-03-1	Nursery, Spain
*Neofusicoccum luteum *	CBS110299	Portugal
*Neofusicoccum australe *	Y264-21-1	Nursery, Spain
*Phaeoacremonium aleophilum *	CBS631.94b	Italy
*P. aleophilum *	Y082-02-5c	Toro, Spain
*Phaeomoniella chlamydospora *	CBS101359	Italy
*P. chlamydospora *	Y170-03-1	Alicante, Spain
*Cylindrocarpon macrodidymum *	V049-01c	Nursery, Spain
*C. macrodidymum *	Y266-10-1	Nursery, Spain
*C. liriodendri *	Y111-07-2c	Arribes, Spain
*C. liriodendri *	Y262-27-1	Nursery, Spain
*C. olidum *	Y160-23-2	Castilla La Mancha, Spain
*C. olidum *	Y160-57-1a	Castilla La Mancha, Spain
*C. olidum *	Y264-22-1	Nursery, Spain
*C. pauciseptatum *	S018-03-1	Salamanca, Spain
*C. pauciseptatum *	S020-02-2	Salamanca, Spain
*Phomopsis viticola *	Y529-07-1	Nursery, Spain
*P. viticola *	Y264-17-1	Nursery, Spain
*Eutypa lata *	Y249-4-4	Toro, Spain
*Fomitiporia mediterranea *	Y255-14-1	Ribera del Duero, Spain
*Stereum hirsutum *	Y112-29-1	Arribes, Spain
*Cryptovalsa ampelina *	Y231-05-4	Ribera del Duero, Spain
*Cadophora luteoolivacea *	Y160-56-2	Castilla La Mancha, Spain
*Fomitiporella coryophilli *	Y234-11-2	Rueda, Spain
*D. coryli *	Y291-24-1	Tierra de León, Spain
*Eutypella citricola *	810	Barcelona, Spain
*Fusarium oxysporum *	Y239-1-5	Rueda, Spain
*Alternaria solani *	CBS109157	USA
*Acremonium* sp.	Y161-9-2	Alicante, Spain
*Epicoccum* sp.	TP32-1C1	Valladolid, Spain
*Psathyrella *sp.	Y266-4-1	Nursery, Spain

**Table 2:** SCAR primer sequences used in this study for the conventional PCR detection of *Diplodia seriata*.

Target	Name	Type	Sequence
Fragment 3.8	DS3.8 S3	Forward primer	5′-ATCCTCATACTACGGCACGG-3′
	DS3.8 R4	Reverse primer	5′-CCGTAGTCTCCCCTTTCCTC-3′
	DS3.8 R6	Reverse primer	5′-AACGGTGACCCATTCCAC-3′

**Table 3:** Detection of *Diplodia seriata* in wood chips.

	Wood chips	Purified DNA		2 days of incubation			
		DS3.8 S3-DS3.8 R6	DS3.8 S3-DS3.8 R4	ME		Water	
				DS3.8 S3-DS3.8 R6	DS3.8 S3-DS3.8 R4	DS3.8 S3-DS3.8 R6	DS3.8 S3-DS3.8 R4
Inoculated vines	Y103-4-2a-1	+	+	+	+	−	−
	Y103-4-2a-2	+	+	−	−	−	−
	Y103-4-2a-3	+	+	+	+	−	−
	Y103-4-2a-4	+	+	+	+	−	−
	Y207-1-1c-1	+	+	−	−	−	−
	Y207-1-1c-2	+	+	+	+	+	+
	Y207-1-1c-3	+	+	+	+	+	+
	Y207-1-1c-4	+	+	+	+	−	−
	PDA-1	−	−	−	−	−	−
	PDA-2	−	−	−	−	−	−
	PDA-3	−	−	−	−	−	−
	PDA-4	−	−	−	−	−	−

Successful reaction		100%	100%	75%	75%	25%	25%

Naturally infected vines	BDA-1	+	+	−	−	−	−
	BDA-2	+	+	+	+	+	+
	BDA-3	+	+	+	+	−	−
	BDA-4	+	+	+	+	+	+
	E. dieback-1	+	+	+	+	−	−
	E. dieback-2	+	+	+	+	+	+
	E. dieback-3	+	+	−	−	−	−
	E. dieback-4	+	+	−	−	−	−

Successful reaction		100%	100%	62.5%	62.5%	37.5%	37.5%

BDA: black dead arm; E. dieback: Eutypa dieback. +: positive amplification of the expected fragment, with DS3.8 S3-DS3.8 R6 (634 bp) and with DS3.8 S3-DS3.8 R4 (233 bp). **−**: no amplification.

## References

[1] Savocchia S., Steel C. C., Stodart B. J., Somers A. (2007). Pathogenicity of *Botryosphaeria* species isolated from declining grapevines in sub tropical regions of Eastern Australia. *Vitis*.

[2] Martin M. T., Cobos R. (2007). Identification of fungi associated with grapevine decline in Castillo y León (Spain). *Phytopathologia Mediterranea*.

[3] Úrbez-Torres J. R., Adams P., Kamas J., Gubler W. D. (2009). Identification, incidence, and pathogenicity of fungal species associated with grapevine dieback in Texas. *The American Journal of Enology and Viticulture*.

[4] van Niekerk J. M., Crous P. W., Groenewald J. Z., Fourie P. H., Halleen F. (2004). DNA phylogeny, morphology and pathogenicity of *Botryosphaeria* species on grapevines. *Mycologia*.

[5] Taylor A., Hardy G. E. S. J., Wood P., Burgess T. (2005). Identification and pathogenicity of *Botryosphaeria* species associated with grapevine decline in Western Australia. *Australasian Plant Pathology*.

[6] Crous P. W., Slippers B., Wingfield M. J. (2006). Phylogenetic lineages in the Botryosphaeriaceae. *Studies in Mycology*.

[7] Phillips A. J. L., Crous P. W., Alves A. (2007). *Diplodia seriata*, the anamorph of *“Botryosphaeria” obtusa*. *Fungal Diversity*.

[8] de Wet J., Slippers B., Preisig O., Wingfield B. D., Wingfield M. J. (2008). Phylogeny of the Botryosphaeriaceae reveals patterns of host association. *Molecular Phylogenetics and Evolution*.

[9] Alves A., Phillips A. J. L., Henriques I., Correia A. (2005). Evaluation of amplified ribosomal DNA restriction analysis as a method for the identification of *Botryosphaeria* species. *FEMS Microbiology Letters*.

[10] McDermott J. M., Brändle U., Dutly F. (1994). Genetic variation in powdery mildew of barley: development of RAPD, SCAR, and VNTR markers. *Phytopathology*.

[11] Délye C., Corio-Costet M.-F. (1998). Origin of primary infections of grape by *Uncinula necator*: RAPD analysis discriminates two biotypes. *Mycological Research*.

[12] Gell I., Cubero J., Melgarejo P. (2007). Two different PCR approaches for universal diagnosis of brown rot and identification of *Monilinia* spp. in stone fruit trees. *Journal of Applied Microbiology*.

[13] Lim S. H., Kim J. G., Kang H. W. (2009). Novel SCAR primers for specific and sensitive detection of *Agrobacterium vitis* strains. *Microbiological Research*.

[14] Pollastro S., Dongiovanni C., Abbatecola A., de Guido M. A., de Miccolis Angelini R. M., Faretra F. (2001). Specific SCAR primers for fungi associated with wood decay of grapevine. *Phytopathologia Mediterranea*.

[15] Lardner R., Stummer B. E., Sosnowski M. R., Scott E. S. (2005). Molecular identification and detection of *Eutypa lata* in grapevine. *Mycological Research*.

[16] Martín L., Sáenz de Miera L. E., Martín M. T. (2014). AFLP and RAPD characterization of *Phaeoacremonium aleophilum* associated with *Vitis vinifera* decline in Spain. *Journal of Phytopathology*.

[17] Cobos R., Martin M. T. (2008). Molecular characterisation of *Phaeomoniella chlamydospora* isolated from grapevines in Castilla y León (Spain). *Phytopathologia Mediterranea*.

[18] Denman S., Crous P. W., Taylor J. E., Kang J., Pascoe I., Wingfield M. J. (2000). An overview of the taxonomic history of *Botryosphaeria*, and a re-evaluation of its anamorphs based on morphology and ITS rDNa phylogeny. *Studies in Mycology*.

[19] Denman S., Crous P. W., Groenewald J. Z., Slippers B., Wingfield B. D., Wingfield M. J. (2003). Circumscription of *Botryosphaeria* species associated with Proteaceae based on morphology and DNA sequence data. *Mycologia*.

[20] Barber P. A., Burgess T. J., Hardy G. E. S. J., Slippers B., Keane P. J., Wingfield M. J. (2005). Botryosphaeria species from Eucalyptus in Australia are pleoanamorphic, producing Dichomera synanamorphs in culture. *Mycological Research*.

[21] Liu J.-K., Phookamsak R., Doilom M. (2012). Towards a natural classification of *Botryosphaeriales*. *Fungal Diversity*.

[22] Martín M. T., Cobos R., Martín L., López-Enríquez L. (2012). Real-Time PCR Detection of *Phaeomoniella chlamydospora* and *Phaeoacremonium aleophilum*. *Applied and Environmental Microbiology*.

